# The triad of collagen, vitamin C, and vitamin E in aging: emerging roles in mood and psychological health, neurotrophic support, cognitive function, endurance, and sarcopenia

**DOI:** 10.3389/fnut.2026.1806863

**Published:** 2026-05-21

**Authors:** Chunzi Xiong

**Affiliations:** School of Humanities and Law, Human Open University, Changsha, Hunan, China

**Keywords:** cognitive outcomes, collagen, elderly subjects, immune, redox balance, sarcopenia, vitamin C, vitamin E

## Abstract

Aging is correlated with a progressive deterioration in muscle mass, strength, metabolic efficiency, vascular and hepatic functions, immune competence, and cognitive capabilities, predominantly influenced by augmented oxidative stress and compromised anabolic signaling pathways. Prophylactic nutritional interventions, particularly those involving collagen, vitamin C, and vitamin E, have emerged as promising, integrative modulators of these age-related declines, especially when combined with structured exercise regimens. Collagen supplementation delivers critical amino acids that facilitate muscle protein synthesis (MPS) and promote tendon integrity, while vitamin C not only enhances collagen biosynthesis but also demonstrates antioxidant and immunomodulatory properties. Vitamin E, recognized as a lipid-soluble antioxidant, serves to safeguard cellular membranes from oxidative damage induced by exercise and plays a significant role in muscle recovery and vascular health. It should be noted that most current evidence examines single nutrients in isolation rather than the integrated triad, limiting the mechanistic clarity of multi-system interactions. This review synthesizes contemporary evidence derived from randomized controlled trials and preclinical investigations examining the synergistic effects of collagen, vitamin C, and vitamin E in conjunction with various exercise modalities as a preventive strategy in elderly cohorts, rather than a therapeutic treatment for established sarcopenia. This discourse examines the outcomes pertinent to skeletal muscle mass, strength capabilities, oxidative stress levels, immune functionality, vascular and hepatic wellness, in addition to cognitive performance metrics. Collectively, the triadic components appear to confer synergistic advantages by facilitating MPS, alleviating oxidative stress, maintaining immune equilibrium, and augmenting metabolic and cognitive resilience among the geriatric population. Future research should emphasize stratification by population characteristics, baseline nutritional status, and exercise modality to clarify differential responses, and should investigate optimal dosing regimens, timing considerations, and mechanistic interactions of the triad with exercise to maximize functional outcomes in older adults.

## 1 Introduction

Aging is characterized by progressive declines across various physiological systems, which encompass skeletal muscle, metabolic regulation, vascular integrity, hepatic function, immune competence, redox homeostasis, and cognitive performance. These modifications are intricately interconnected and are propelled by common mechanisms, including chronic low-grade inflammation, mitochondrial dysfunction, impaired extracellular matrix (ECM) remodeling, and cumulative oxidative stress (1–4). Sarcopenia, insulin resistance, endothelial dysfunction, immunosenescence, and cognitive impairment are frequently observed in the elderly population, collectively exacerbating frailty, disability, and the loss of autonomy (5, 6). Despite an increasing acknowledgment of these interrelations, the majority of therapeutic approaches remain organ-specific, neglecting to consider aging as a comprehensive systems-level phenomenon. Nutritional and lifestyle modifications represent a promising preventive strategy to influence these interrelated biological pathways, although the precise long-term efficacy of specific nutrient combinations, such as collagen, vitamin C, and vitamin E, remains incompletely established due to limited integrative and longitudinal studies (7). Among the dietary components, collagen, vitamin C, and vitamin E form a biologically coherent yet inadequately integrated triad. Collagen is critical not only for maintaining musculoskeletal integrity but also for ensuring vascular elasticity, hepatic architecture, and the structural framework of neural extracellular matrices (8, 9). Vitamin C is essential for the hydroxylation of collagen, immune defense mechanisms, and the regulation of redox homeostasis (10), whereas vitamin E functions as a principal lipid-soluble antioxidant that safeguards cellular membranes, mitochondria, and neural tissues from oxidative damage (11, 12). However, empirical support for the synergistic benefits of these nutrients when combined with exercise remains largely inferred, as most studies have examined them individually rather than as a coordinated intervention, and caution should be exercised when extrapolating short-term biomarker changes to long-term functional outcomes.

Exercise serves as a pivotal modulator of healthy aging and a fundamental determinant of the manner in which nutritional interventions affect physiological outcomes within a preventive and health-promoting context rather than as a clinical treatment strategy (13, 14). Engaging in physical activity orchestrates the regulation of collagen turnover, mitochondrial biogenesis, the expression of antioxidant enzymes, immune surveillance, and neuroplasticity (15). Of considerable importance, reactive oxygen species (ROS) generated through exercise act as signaling molecules essential for adaptation, thereby raising concerns that indiscriminate supplementation with antioxidants may attenuate advantageous training responses (16, 17). Nevertheless, this discourse has infrequently distinguished between the excess of pharmacological antioxidants and the physiological, context-dependent synergy achieved through dietary-level intake aimed at supporting healthy aging and functional maintenance, rather than treating established pathological conditions such as sarcopenia. Moreover, inconsistencies in the literature, including the limited anabolic effect of collagen relative to high-quality proteins and the potential for high-dose antioxidants to blunt training adaptations, underscore the need for careful, critical appraisal and caution in generalizing results. Significantly, the synergistic effects of collagen, vitamin C, and vitamin E in conjunction with exercise especially among elderly populations, have yet to be systematically synthesized. To this point, no literature review has thoroughly synthesized the structural (collagen), biochemical (vitamin C), and lipid-protective (vitamin E) mechanisms across various domains including muscular, metabolic, vascular, hepatic, immune, oxidative, and cognitive systems, while distinctly positioning exercise as a modulating factor rather than as a confounding variable. Current reviews predominantly concentrate on individual nutrients, isolated antioxidant effects, or specific outcomes such as sarcopenia or oxidative stress, thereby creating a substantial conceptual void in the comprehension of how integrated interventions may affect the trajectories of aging (18–20).

The objective of the current review is to rigorously assess the collagen–vitamin C–vitamin E triad as a multifaceted modulator of aging-related outcomes, placing particular focus on its interplay with physical activity. We consolidate mechanistic evidence, highlight discrepancies, and contextualize findings within the paradigm of physiological aging and preventive, non-therapeutic nutritional strategies for healthy aging. Central themes arising from existing knowledge and empirical experience indicate that aging is intrinsically systemic, that this triad operates as a mutually dependent biological axis, and that the context of exercise critically influences whether antioxidant and structural support yields beneficial or detrimental effects. It is important to note that this triad is primarily considered as a prophylactic measure to maintain muscle mass, strength, and overall function, rather than a proven therapeutic intervention for established sarcopenia, and that most evidence derives from short-term or biomarker-based studies rather than longitudinal functional outcomes. Future investigations should transcend reductionist, single-nutrient methodologies in favor of exercise-integrated, multi-component interventions, underpinned by biomarker-guided personalization strategies. Robustly powered longitudinal studies are necessary to clarify the precise synergistic effects, optimal dosages, timing relative to exercise, and population-specific responses, particularly with respect to long-term functional and cognitive outcomes. Methodologically robust longitudinal human studies encompassing functional, molecular, and cognitive endpoints are imperative to ascertain optimal dosing, timing, and population-specific responses. Such advancements are crucial for translating mechanistic insights into pragmatic strategies that sustain function, resilience, and autonomy in aging populations through preventive nutritional and lifestyle interventions.

## 2 Aging, oxidative stress, and inflammation

### 2.1 Mechanisms of oxidative stress and chronic low-grade inflammation in aging

**Figure 1:**
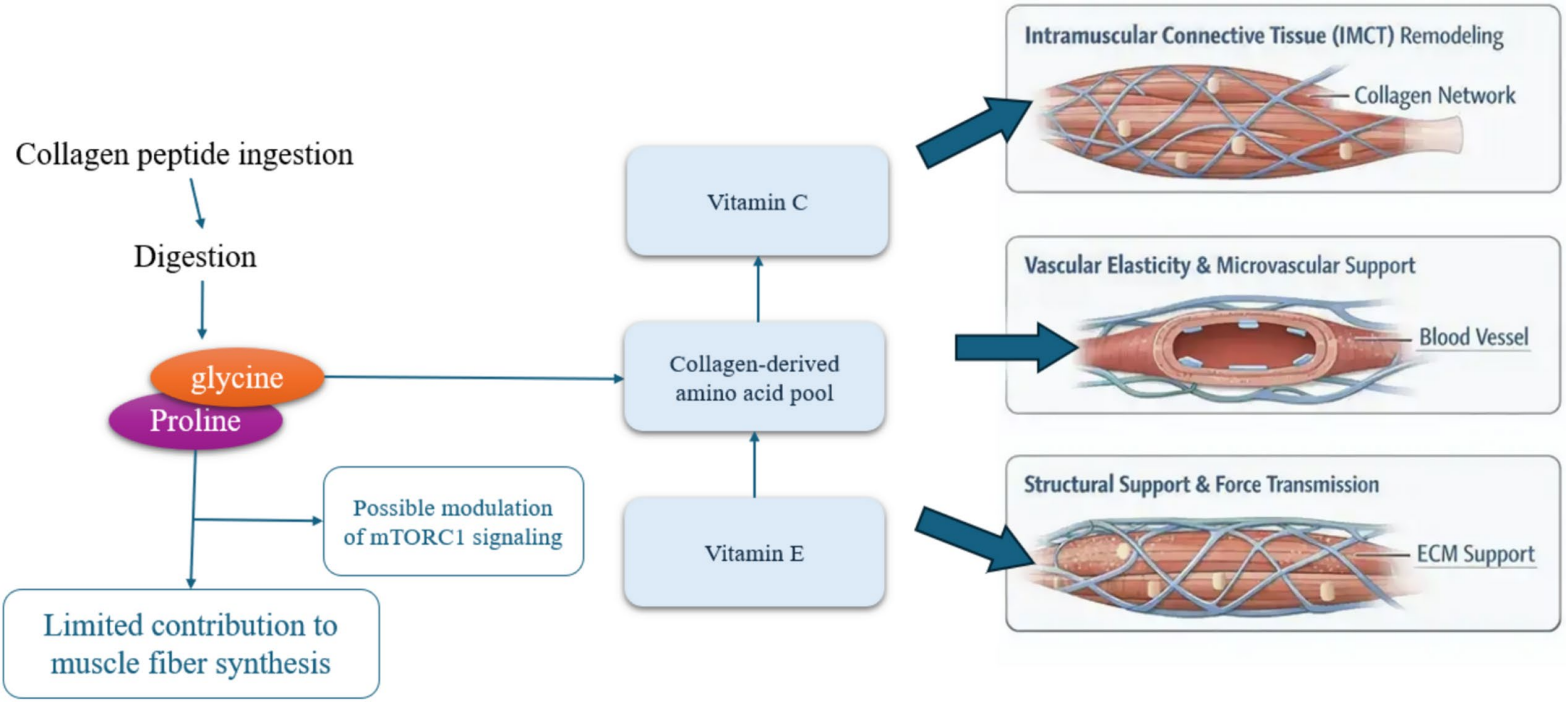
Conceptual model illustrating the role of collagen-derived amino acids in supporting intramuscular connective tissue and vascular elasticity within skeletal muscle. Following ingestion, collagen peptides are digested into characteristic amino acids and dipeptides, particularly glycine, proline, and hydroxyproline. These components are incorporated into the extracellular matrix (ECM) of skeletal muscle, contributing to the maintenance and remodeling of intramuscular connective tissue (IMCT), including the endomysium, perimysium, and epimysium. Strengthening of this connective tissue network supports force transmission, structural integrity, and mechanical resilience of muscle fibers. Collagen-derived amino acids also contribute to vascular connective tissue, supporting the structural properties and elasticity of blood vessels that supply skeletal muscle. Together, these processes help maintain the functional microenvironment of muscle tissue. Although amino acid availability can influence nutrient-sensitive signaling pathways such as mTORC1, collagen peptides are not considered primary drivers of myofibrillar protein synthesis. Instead, their physiological contribution is primarily associated with connective tissue support, extracellular matrix remodeling, and vascular elasticity, which indirectly support skeletal muscle function and adaptation.

Aging is delineated by a gradual disruption in the equilibrium between the synthesis of ROS and the efficacy of antioxidant defense mechanisms, resulting in a persistent condition of oxidative stress (21). Mitochondrial dysfunction serves as a pivotal catalyst in this phenomenon, as age-associated deficits in the efficiency of the electron transport chain escalate electron leakage and ROS production (1). Simultaneously, antioxidant enzymes such as superoxide dismutase, catalase, and glutathione peroxidase exhibit diminished activity or dysregulated expression as chronological age progresses. Oxidative stress further exacerbates cellular damage through mechanisms such as lipid peroxidation, protein carbonylation, and DNA oxidation, thereby reinforcing a self-sustaining cycle of mitochondrial deterioration. Intricately linked to oxidative stress is the phenomenon of chronic low-grade inflammation, commonly referred to as inflammaging. This pathological state emerges from the sustained activation of innate immune mechanisms, an elevated burden of senescent cells, and the dysregulation of redox-sensitive signaling pathways, including the nuclear factor kappa B and NLRP3 inflammasome activation (2, 22). Senescent cells release a variety of pro-inflammatory cytokines, chemokines, and proteolytic enzymes collectively designated as the senescence-associated secretory phenotype, which further intensify oxidative injury and compromise tissue integrity. Crucially, oxidative stress and inflammation are not solely the byproducts of the aging process, but rather active elements that facilitate functional deterioration across diverse organ systems, thereby establishing a common molecular substrate for age-related diseases instead of distinct pathological entities (Figure 1).

### 2.2 Implications for muscle, vascular, hepatic, immune, and cognitive health

The intersection of oxidative stress and persistent inflammation engenders significant multisystem repercussions in the context of aging. Within skeletal muscle, the accumulation of ROS disrupts mitochondrial biogenesis, hinders calcium homeostasis, and stimulates proteolytic pathways, including the ubiquitin–proteasome and autophagy–lysosome systems, thereby accelerating sarcopenia and functional deterioration (23, 24). Vascular tissues exhibit comparable susceptibility, as oxidative stress diminishes nitric oxide (NO) bioavailability, exacerbates endothelial dysfunction, and promotes arterial stiffness and atherosclerosis via redox-sensitive inflammatory signaling mechanisms (25). In the hepatic tissue, age-associated oxidative stress plays a pivotal role in the dysregulation of lipid metabolism, mitochondrial dysfunction, and an increased vulnerability to non-alcoholic fatty liver disease, which occurs in part through inflammatory interactions with adipose tissue and the gut-liver axis. The immune system experiences a concomitant decline, characterized by inflammaging, which exacerbates immunosenescence, diminishes pathogen clearance, and leads to compromised vaccine efficacy (26). Within the central nervous system, sustained oxidative and inflammatory stress undermines the integrity of the blood–brain barrier, disrupts synaptic plasticity, and hastens neurodegenerative processes that contribute to cognitive decline and dementia (1, 27, 28). The aforementioned modifications collectively emphasize that oxidative stress and inflammation operate as systemic integrators of the biological processes associated with aging, thereby establishing connections among structural decline, metabolic irregularities, and cognitive deficits. This interrelated susceptibility accentuates the necessity for interventions such as specific prophylactic nutritional strategies and physical activity, that aim to restore redox equilibrium while facilitating adaptive signaling, rather than indiscriminately inhibiting oxidative mechanisms.

## 3 Collagen supplementation and exercise in older adults

### 3.1 Role of collagen in muscle protein synthesis and musculoskeletal integrity

**Figure 2:**
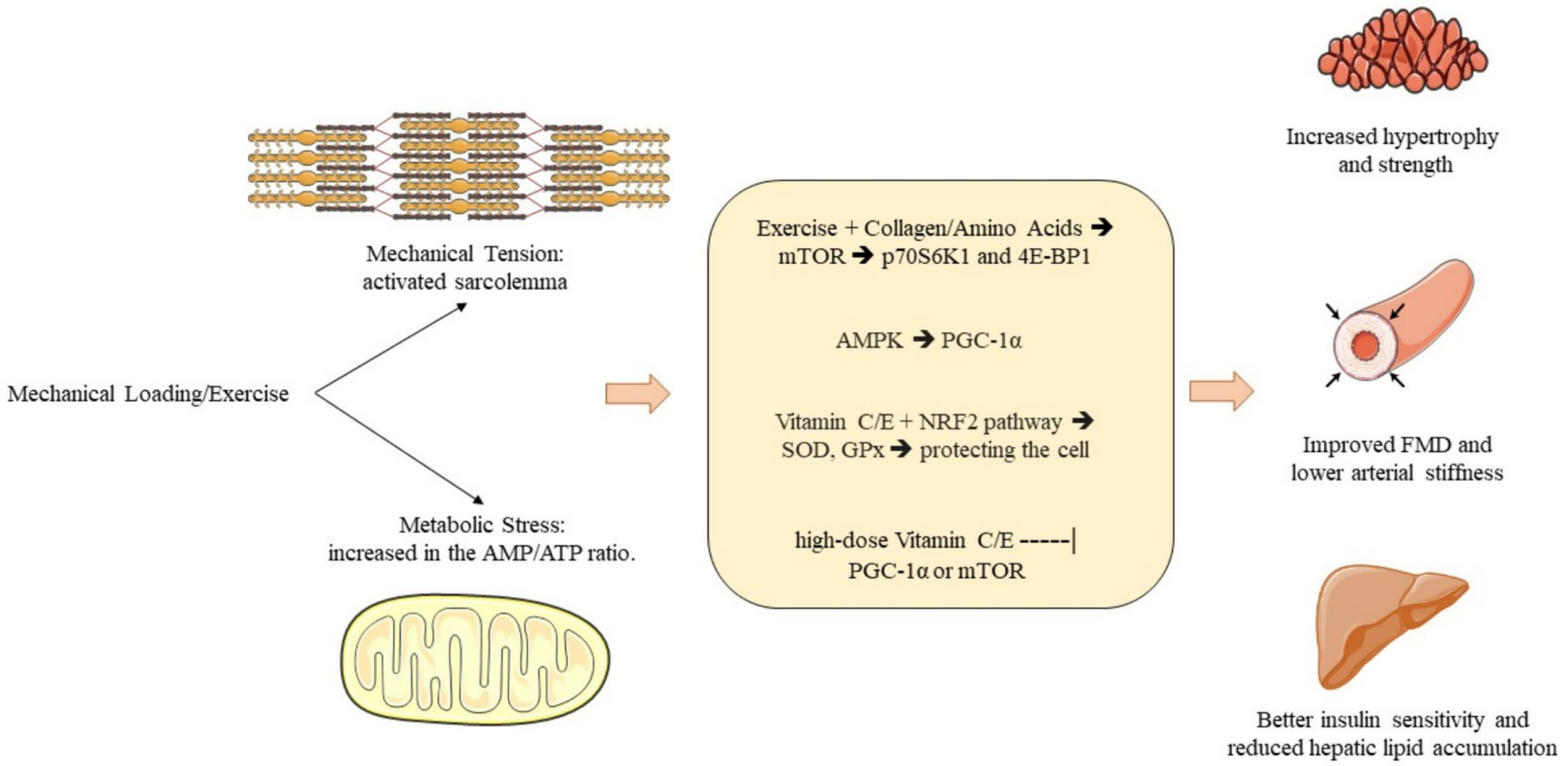
Integrated pathways of exercise-induced adaptations and nutritional synergy. Mechanical loading and amino acid availability (from collagen) converge on the mTORC1–p70S6K1 pathway to stimulate muscle protein synthesis (MPS). Concurrently, metabolic stress activates the AMPK–PGC-1alpha axis, driving mitochondrial biogenesis and aerobic capacity. Vitamins C and E support these processes by modulating the Nrf2 redox response and protecting against excessive exercise-induced ROS. Supra-physiological doses of antioxidants may theoretically blunt the hormetic ROS signals (dashed T-bar) required for optimal PGC-1alpha activation and vascular FMD improvements.

Collagen, recognized as the predominant structural protein within the human body, plays a pivotal role in the formation of the ECM in skeletal muscle and connective tissues. In the elderly population, the phenomena of age-associated anabolic resistance and sarcopenia significantly impair the ability for muscle protein synthesis (MPS) to respond to conventional anabolic stimuli, such as resistance exercise and the intake of high-quality protein, which can be attributed, in part, to a deterioration in muscle quality and alterations in ECM structure as one ages. Although research indicates that supplemental collagen peptides can lead to a modest increase in muscle mass and strength when utilized in conjunction with resistance training (RT) in older men experiencing sarcopenia, the specific effects of collagen on MPS remain ambiguous, likely due to its relatively low leucine concentration in comparison to other protein sources, such as whey. Certain investigations propose that collagen supplementation may not substantially promote myofibrillar protein synthesis in older adults when adjusted for protein content, highlighting the critical need for comprehensive nutrition and exercise approaches to maintain musculoskeletal health in the aging population (29–32) (Figure 2). In the realm of sarcopenia, it is essential to differentiate between the regulation of MPS and the remodeling of the ECM, as these biological processes exhibit distinct responses to nutritional stimuli. Collagen is notably characterized by a substantial concentration of glycine, proline, and hydroxyproline; however, it is deficient in essential amino acids, particularly leucine, which serves as a pivotal activator of the mTORC1 signaling pathway and functions as a principal initiator of MPS. As a result, the supplementation of collagen in isolation is improbable to optimally induce myofibrillar hypertrophy, particularly in older adults who manifest anabolic resistance to protein consumption (33, 34). Conversely, leucine-enriched protein sources, such as whey protein and branched-chain amino acids, have been consistently demonstrated to effectively stimulate MPS and facilitate increases in muscle mass and strength, thus rendering them more appropriate for addressing the loss of contractile proteins associated with age-related sarcopenia (35). Nevertheless, the functional capacity of skeletal muscle is not exclusively reliant on myofibrillar proteins; it is also significantly influenced by the structural integrity and mechanical characteristics of the ECM, which is pivotal in force transmission, elasticity, and the prevention of injuries. In this context, collagen supplementation may provide advantageous effects by promoting the remodeling of connective tissue, augmenting tendon stiffness, and reinforcing musculoskeletal integrity, particularly when synergistically administered with vitamin C, a vital component for the synthesis of collagen (36, 37). Consequently, although collagen may be suboptimal for the direct augmentation of myofibrillar mass, it has the potential to complement leucine-rich proteins by focusing on the non-myofibrillar constituents of muscle tissue. In summary, an integrated nutritional strategy may prove to be the most efficacious in addressing sarcopenia, where the adequate consumption of high-quality, leucine-rich proteins is emphasized to stimulate MPS, while collagen supplementation especially in conjunction with vitamin C and potentially vitamin E for its antioxidant benefits, facilitates ECM remodeling and enhances overall musculoskeletal functionality. This dual-target strategy aligns with the multifactorial nature of sarcopenia and highlights the complementary rather than substitutive role of collagen within the proposed nutritional triad.

### 3.2 Effects on tendon, joint, and metabolic outcomes

Collagen supplementation, particularly when administered alongside structured physical training, may provide advantages that extend beyond mere augmentation of muscle mass by promoting the health of connective tissues in the geriatric population. Empirical investigations suggest that a daily intake of collagen peptides, in conjunction with RT, can lead to enhanced body composition, characterized by an increase in fat-free mass and a reduction in fat mass among elderly individuals suffering from sarcopenia, and may also facilitate improvements in joint functionality and alleviation of pain, likely through the enhancement of ECM synthesis within connective tissues. The contribution of collagen to the optimization of tendon morphology and adaptations of musculotendinous structures has been documented in systematic reviews, revealing tendencies toward an increased cross-sectional area of tendons and alterations in mechanical properties when amalgamated with training regimens. Furthermore, collagen hydrolysates have been correlated with enhanced joint mobility and diminished degenerative symptoms among older adults, potentially attributable to an increase in the synthesis of connective proteins and a reduction in their degradation. Additionally, certain evidence indicates improvements in metabolic function, underscoring the multifaceted impacts of collagen on both musculoskeletal and metabolic health in older adults when integrated with exercise interventions (29, 38, 39).

### 3.3 Evidence from human and preclinical studies

Aging is correlated with progressive reductions in skeletal muscle mass, strength, and functional capacity, which significantly elevate the risk of sarcopenia, frailty, and loss of autonomy among older adults. Resistance exercise is a well-documented intervention to mitigate these alterations; however, the phenomenon of anabolic resistance constrains the efficacy of exercise in isolation, underscoring the necessity for optimized prophylactic nutritional strategies. Collagen supplementation has garnered increasing interest as a potentially accessible and well-tolerated protein source for geriatric populations, yet its efficacy in comparison to high-quality proteins and its interaction with various exercise modalities remain subjects of contention. This review elucidates the role of collagen-based supplementation as a preventive intervention when integrated with resistance or blood flow restriction (BFR) training in older adults, offering mechanistic and functional insights into muscle hypertrophy, strength adaptations, and metabolic responses. By contrasting collagen with alternative protein sources and exercise methodologies, these findings contribute to the refinement of evidence-based preventive nutritional guidelines and highlight the limitations of collagen supplementation in maintaining musculoskeletal health throughout the aging process. A clinical trial investigated the impact of collagen peptide supplementation combined with 12 weeks of supervised RT on body composition and muscle function in elderly sarcopenic men. A total of 53 sarcopenic males (with a mean age of approximately 72 years) engaged in a 12-week supervised RT regimen (comprising three sessions per week) and were administered either 15 g/day of collagen peptides or a placebo. Variations in fat-free mass, fat mass, and bone mass were measured through dual-energy X-ray absorptiometry, while muscular functionality was assessed utilizing isokinetic quadriceps strength tests and sensory motor control evaluations. RT yielded significant enhancements in body composition and neuromuscular performance across all participants. Nevertheless, collagen peptide supplementation led to more substantial increases in fat-free mass and quadriceps strength, in addition to a more marked decrease in fat mass, in comparison to the placebo group. Enhancements in sensory motor control and bone mass were likewise noted subsequent to the intervention (20) (Table 1). A long-term study evaluated the effect of one-year protein supplementation, with or without exercise, on MPS and the skeletal muscle metabolome in healthy older adults. Sixty-six participants, each aged 65 years or older, were systematically distributed into five intervention cohorts receiving either carbohydrate, collagen protein, whey protein, whey protein in conjunction with light RT, or whey protein in combination with heavy-load RT for a duration of 12 months. Basal and postprandial MPS were evaluated utilizing stable isotope tracer infusion prior to and subsequent to the intervention, following the consumption of a standardized whey hydrolysate–glucose mixture. The skeletal muscle metabolomic profiles were scrutinized through gas chromatography–mass spectrometry at both baseline and postprandial time points. Following the one-year period, neither protein nor carbohydrate supplementation, irrespective of exercise engagement, produced significant modifications in basal or postprandial MPS or muscle metabolomic profiles. Baseline MPS rates exhibited no significant differences between genders, and untargeted metabolomic analysis revealed the presence of over 70 muscle metabolites (40).

**Table 1:** Effects of collagen supplementation combined with exercise on muscle mass, strength, and metabolic outcomes in older adults.

Study population	Study population	Exercise protocol	Main findings	References
Elderly men with sarcopenia (72.2 ± 4.68 years)	Collagen peptides (15 g/day) Placebo	Supervised resistance training, 3×/week	FFM ↑↑ (greater with collagen vs. placebo) IQS ↑↑ (greater with collagen) FM ↓↓ (greater reduction with collagen) BM ↑ SMC ↑	(20)
Healthy older adults (>65 years)	Carbohydrate Collagen protein Whey protein Whey + light RT Whey + heavy RT	Light or heavy resistance training	Basal MPS ↔ Postprandial MPS ↔ Skeletal muscle metabolome ↔ Sex differences in MPS ↔	(40)
Healthy older adults (≥65 years)	Whey protein (3.1 μg B12/day) Collagen (1.3 μg B12/day) Carbohydrate	Heavy RT, light RT, or no training	Plasma vitamin B12 ↑ (whey only) Holotranscobalamin ↑ (whey only) Methylmalonic acid ↓ (light RT + whey) B12 biomarkers ↔ (collagen, carbohydrate) Training regimen ↔ (no independent effect)	(41)
Premenopausal women	Specific collagen peptides (15 g/day) Placebo	Resistance training, 3×/week for 12 weeks	Fat-free mass ↑↑ (greater with collagen) Body fat ↓↓ (greater with collagen) Hand-grip strength ↑ (greater with collagen) Leg strength ↑↑ (greater with collagen)	(42)
Healthy older women (69 ± 3 years)	Whey protein (30 g, twice daily) Collagen protein (30 g, twice daily)	Unilateral resistance exercise (acute and 6-day protocol)	Acute MPS ↑↑ (whey, rest + exercise) Acute MPS ↑ (collagen, exercise only) Longer-term MPS ↑↑ (whey, rest + exercise) Longer-term MPS ↔ (collagen) MPS response: whey > collagen	(43)
Aged male mice (24 months)	Enriched collagen peptide (GPT-2218) Generic fish collagen Standard diet	Endurance treadmill training	VO₂max ↑ (GPT-2218 + training) Muscle strength ↑ (GPT-2218 + training) Oxidative stress markers ↓ (faster recovery with GPT-2218) Inflammatory balance ↑ (IL-10 ↑, IL-1β ↓ recovery) Skeletal muscle glycogen preservation ↑ (GPT-2218) Generic fish collagen ↔	(44)
Older men; overweight/obese with metabolic syndrome risk	Collagen-based MIS (PLA) Whey/casein-based MIS (M5)	Home-based resistance training + daily physical activity	Obesity/MetS → anabolic response ↓↓ Lean mass gains ↓ with higher MetS risk Lean mass ↑ (M5 > collagen) Strength ↑ (leg press; M5 only) Physical performance ↑ (stair-climb time ↓; M5 only) Bone turnover markers ↑ (M5 only)	(45)
Healthy older men (60.1 ± 7.6 years)	BFR training + collagen hydrolysate BFR training + placebo Protein only (no training)	Low-load blood-flow restriction training	Muscle CSA ↑↑ (BFR, with or without collagen) Muscle strength ↑ (time effect) IGF-1 ↔ Reactive oxygen species ↔ Added collagen effect ↔	(46)

Symbols indicate the statistical significance of the changes reported in the original studies.

↑↑/↓↓: highly significant increase or decrease (*p* < 0.001).

↑/↓: significant increase or decrease (*p* < 0.05).

↔: no significant change (*p* > 0.05).

Extending the nutritional focus, another year-long intervention examined the effect of protein supplementation and RT on vitamin B12 biomarkers in older adults. A total of 167 subjects were allocated to receive bi-daily supplements of whey protein, collagen protein, or maltodextrin, with the whey protein cohort further divided into heavy RT, light RT, or no-training subgroups. Dietary consumption was systematically tracked through 3-day food diaries, and longitudinal evaluations encompassed 110 participants who satisfied predetermined compliance criteria. The biomarkers evaluated included plasma vitamin B12, holotranscobalamin, and methylmalonic acid, which were quantified under both fasting and non-fasting conditions at various time intervals. Whey protein supplementation was found to significantly elevate circulating concentrations of vitamin B12 and holotranscobalamin, with a noteworthy reduction in methylmalonic acid observed among the light RT subgroup. Conversely, the supplementation of collagen and carbohydrate intake did not result in any modifications to vitamin B12 biomarkers, and RT in isolation exhibited no independent effects (41). A parallel study investigated specific collagen peptide supplementation in premenopausal women undergoing RT. A total of 77 subjects participated in a 12-week supervised RT regimen, conducted three times weekly, and were assigned to receive either 15 g/day of specific collagen peptides or a placebo. Alterations in body composition were evaluated employing bioelectrical impedance analysis, while muscular strength was quantified through isometric strength assessments, incorporating hand-grip and leg strength measurements. Both cohorts demonstrated significant enhancements in fat-free mass and decreases in body fat subsequent to RT. Nevertheless, the administration of collagen peptides yielded a statistically significant augmentation in fat-free mass and a more marked reduction in fat mass relative to the placebo group. Furthermore, improvements in hand-grip and leg strength were recorded in both groups, with participants receiving collagen peptide supplementation exhibiting substantially greater advancements (42).

Further probing protein quality, a six-day intervention in older women compared whey versus collagen ingestion combined with resistance exercise. A total of 22 subjects (average age 69 years) ingested 30 g of either whey protein or collagen protein bi-daily over a span of 6 days. Unilateral resistance exercise was administered twice throughout the intervention to assess conditions of feeding-only and feeding-plus-exercise. Acute MPS was quantified utilizing [^13^C₆]-phenylalanine infusion, while prolonged MPS was evaluated through the consumption of deuterated water. Whey protein significantly augmented acute MPS in both resting and exercised muscle segments, whereas collagen protein enhanced MPS exclusively post-exercise. Prolonged MPS saw a significant increase with whey protein under both conditions, yet remained invariant with collagen supplementation. Across both acute and prolonged assessments, MPS responses were consistently superior following whey protein consumption in comparison to collagen protein (43). In aged mice, the combination of enriched collagen peptide (GPT-2218) supplementation and endurance exercise was examined for effects on exercise performance and metabolic homeostasis. Mice aged 24 months were allocated to either standard or supplemented dietary regimes and subsequently subdivided into sedentary or treadmill training cohorts, culminating in six distinct experimental conditions, which included a comparator group receiving generic fish collagen peptides. Endurance training was conducted over a two-week period at intensities approximating 75% of maximal oxygen uptake. The measured outcomes encompassed VO₂max, muscular strength, hepatic and skeletal muscle glycogen concentrations, oxidative stress indicators, activity levels of antioxidant enzymes, and inflammatory cytokines. The supplementation of GPT-2218 in conjunction with training markedly improved VO₂max, forelimb strength, antioxidant recovery, resolution of inflammation, and post-exercise glycogen replenishment relative to training conducted in isolation. Conversely, supplementation with generic fish collagen yielded no discernible effects across physiological, metabolic, or inflammatory metrics (44).

A retrospective analysis assessed predictors of adaptive response to home-based RT plus multi-ingredient protein supplementation in older men, including collagen and whey/casein-based formulas. A total of 32 subjects were scrutinized to ascertain baseline predictors of variations in lean mass, body composition metrics, strength, and physical performance. The status of obesity and a composite index of metabolic syndrome risk were identified as the most significant negative predictors of enhancements in total and appendicular lean mass, lean mass-to-fat mass ratios, and allometric lean mass metrics. Subgroup analyses involving overweight or obese individuals possessing additional metabolic risk factors compared the efficacy of a collagen-based supplement with that of a whey/casein-based multi-ingredient formulation. Participants who received the whey/casein-based supplementation displayed more substantial advancements in lean mass indices, lower-body strength, functional performance, and markers of bone turnover indicative of bone formation, while the collagen-based supplement demonstrated limited adaptive responses (45). Finally, an eight-week intervention evaluated post-exercise collagen hydrolysate supplementation with low-load BFR training in older men. A total of 30 participants (mean age approximately 60 years) underwent an eight-week intervention, during which they were allocated to either low-load BFR training with collagen hydrolysate supplementation, BFR training with a placebo, or a non-training control group that received protein supplementation. The primary outcomes measured included muscle cross-sectional area, isometric strength, circulating insulin-like growth factor-1, and the production of ROS. Both groups undergoing BFR training demonstrated substantial increases in muscle cross-sectional area, while the control group exhibited no significant alterations. Nevertheless, collagen supplementation did not yield a statistically significant additive effect on muscle hypertrophy when compared to the placebo. Assessments of muscle strength, IGF-1, and oxidative stress indicated significant temporal effects; however, no group-specific interactions were identified, suggesting that collagen hydrolysate did not substantially alter physiological responses to BFR training within this particular cohort (46). Collectively, these studies demonstrate that collagen supplementation can modestly enhance MPS, particularly when paired with mechanical loading, improve neuromuscular adaptations, and support redox and inflammatory balance in aging models. However, whey and other leucine-rich proteins consistently produce stronger anabolic responses, and factors such as obesity, metabolic syndrome, and protein composition significantly influence outcomes. Collagen interacts synergistically with exercise to maintain fat-free mass, strength, and metabolic homeostasis, though its efficacy is context- and load-dependent.

## 4 Vitamin C and exercise: metabolic, immune, and vascular outcomes

### 4.1 Mechanisms of antioxidant and immunomodulatory effects

Vitamin C serves as a highly effective water-soluble antioxidant by facilitating the donation of electrons to neutralize ROS that are generated during both exercise and metabolic processes, thereby safeguarding lipids, proteins, and DNA from oxidative harm. In terms of its mechanisms, ascorbate is actively involved in redox-dependent biochemical reactions and aids in the regeneration of endogenous antioxidants, such as glutathione, which is vital for preserving intracellular redox equilibrium during states of physiological stress and the production of ROS associated with exercise. Furthermore, vitamin C functions as a critical cofactor in various enzymatic processes, including the biosynthesis of carnitine, which is essential for the mitochondrial transport of fatty acids, as well as collagen synthesis, which is crucial for maintaining tissue integrity when subjected to mechanical stress. From the standpoint of immunomodulation, vitamin C has a significant impact on both innate and adaptive immune responses, mitigating exercise-induced oxidative stress and the release of inflammatory cytokines (e.g., IL-6) in acute scenarios, while concurrently enhancing lymphocyte functionality and decreasing infection incidence following exercise. These intricate biochemical pathways elucidate the manner in which vitamin C plays a pivotal role in the cellular defense mechanisms that converge with critical stress responses related to exercise (47, 48).

### 4.2 Impact on muscle function, vascular health, liver metabolism, and immune gene expression

The interaction between vitamin C and physical exercise manifests a plethora of effects across metabolic, immunological, and vascular systems. Empirical evidence from randomized trials indicates that supplementation may mitigate exercise-induced oxidative stress, leading to a reduction in lipid peroxidation and a decrease in pro-inflammatory IL-6 levels subsequent to acute exercise sessions; however, the impact on muscle soreness, strength, and biomarkers such as CK and CRP remains inconsistent. This antioxidative property has the potential to bolster muscle metabolic function by maintaining cellular integrity amidst elevated ROS production. With respect to vascular health, the provision of supplemental vitamin C has been demonstrated to enhance endothelial function and the bioavailability of NO, particularly in populations at risk for cardio-metabolic conditions, thereby improving flow-mediated dilation (FMD) and diminishing arterial stiffness associated with oxidative stress. Furthermore, vitamin C plays a significant role in lipid metabolism and antioxidative defense mechanisms within hepatic models, influencing gene expression pertinent to inflammatory and oxidative pathways when paired with exercise interventions in animal research. The modulation of immune gene expression also intersects with exercise, although the available human data exhibit variability contingent upon the intensity and duration of the intervention (49, 50).

### 4.3 Evidence from human and preclinical studies

A research examined the influence of physical training in conjunction with vitamin C supplementation on iron metabolism among elderly women. A total of 15 participants engaged in a 12-week intervention, comprising 6 weeks of training exclusively and an additional 6 weeks of training supplemented with a daily intake of 1,000 mg of vitamin C. The participants were divided into two cohorts that alternated the timing of vitamin C supplementation: cohort 1 received vitamin C during the initial six-week phase, whereas cohort 2 received it during the subsequent six-week phase. Health-oriented training sessions were conducted thrice weekly throughout the duration of the study. The administration of vitamin C did not significantly modify the plasma prooxidative/antioxidative equilibrium; however, it notably diminished the mRNA expression levels of ferritin heavy chain and ferritin light chain (FTL) within leukocytes. Specifically, the mRNA levels of ferritin heavy chain (FTH) decreased from 2^64.24^ to 2^11.06^ (*p* = 0.03) in cohort 1 and from 2^60.54^ to 2^16.03^ (*p* = 0.01) in cohort 2, while the mRNA levels of FTL decreased from 2^20.22^ to 2^4.53^ (*p* = 0.01) in cohort 2 (51) (Table 2). Another investigation explored the impact of physical exercise in conjunction with vitamin C supplementation on the modulation of inflammation-associated gene expression in elderly female subjects. A total of 24 participants were randomly allocated into a supplemented cohort (SUP, *n* = 12, receiving 1,000 mg of vitamin C per day) or a placebo-controlled cohort (CON, *n* = 12) and engaged in a six-week health enhancement regimen conducted three times weekly. The expression levels of CCL2, CRP, IL1, IL6, and IL10 mRNA in leukocytes were quantified both prior to and subsequent to the intervention. No statistically significant alterations were detected in the mRNA levels of IL-1, IL-6, IL-10, or CRP within or across the experimental groups. Nevertheless, there was an observable trend indicating a decrease in IL-6 and an increase in IL-10 within the supplemented cohort. Notably, a statistically significant reduction in CCL2 mRNA was exclusively observed in the control group (from 2^0.2^ to 2^0.1^, *p* = 0.01), suggesting distinct gene-specific responses to the combined effects of supplementation and physical training (52).

**Table 2:** Effects of vitamin C supplementation combined with exercise on metabolic profiles, immune-related gene expression, muscle, vascular, and hepatic outcomes in elderly subjects.

Study population	Intervention groups	Exercise protocol	Main findings	References
Elderly women	Training + vitamin C (1,000 mg/day) Training alone (cross-over)	Health-oriented training, 3×/week	Ferritin mRNA (FTH, FTL) ↓↓ (vitamin C + training) Prooxidative/antioxidative balance ↔ Systemic oxidative stress ↔	(51)
Elderly women (≈73 years)	Vitamin C (1,000 mg/day) + training Placebo + training	Health-oriented training, 3×/week for 6 weeks	IL-6 mRNA ↓ (trend, vitamin C) IL-10 mRNA ↑ (trend, vitamin C) IL-1 mRNA ↔ CRP mRNA ↔ CCL2 mRNA ↓ (control only)	(52)
Aged male rats on high-fat diet	Vitamin C + silymarin Endurance swimming Vitamin C + silymarin + swimming	Endurance swimming, 5×/week	Muscle fiber diameter ↑ (exercise + supplementation) Muscle atrophy ↓ (combined intervention) mTOR signaling ↓ (HFD) IGF-1, S6K1, 4E-BP1 ↑ (exercise + supplementation) Lipid profile ↑ (HFD)/↓ (exercise + supplementation)	(53)
Young sedentary men; older sedentary men; older endurance-trained men	Acute vitamin C infusion Chronic oral vitamin C (500 mg/day)	Habitual endurance exercise (trained group)	Flow-mediated dilatation (FMD) ↓ (aging, sedentary) FMD ↑ (acute vitamin C, older sedentary) FMD ↔ (chronic vitamin C) Endothelium-independent dilation ↔ Exercise preserved FMD with aging	(54)
Elderly male rats with HFD-induced liver damage	Vitamin C + silymarin Swimming exercise Combined intervention	Swimming, 5×/week	Hepatic TNF-α, IL-1β ↓ (combined intervention) Oxidative stress ↓ Total antioxidant capacity ↑ PPAR-α ↑ Liver fat accumulation ↓	(55)

Symbols indicate the statistical significance of the changes reported in the original studies.

↑↑/↓↓: highly significant increase or decrease (*p* < 0.001).

↑/↓: significant increase or decrease (*p* < 0.05).

↔: no significant change (*p* > 0.05).

The aim of study was to determine the ramifications of an eight-week regimen of endurance swimming concomitant with vitamin C and Silymarin supplementation on skeletal muscle and hepatic outcomes in aged Wistar rats subjected to a high-fat diet (HFD). A total of 25 aged male Wistar rats were systematically allocated into five experimental cohorts: normal diet (Control), HFD, HFD + combined supplementation (HFD + CS), HFD + endurance swimming (HFD + ES), and HFD + CS + ES. Following a six-week exposure to HFD, supplementation was administered via gavage for an additional 8 weeks, while swimming exercise was conducted five times weekly within the exercise cohorts. The HFD condition resulted in an augmented lipid accumulation in the hepatic tissue and precipitated skeletal muscle atrophy. The amalgamation of exercise and supplementation yielded improvements in liver histology, modulated lipid profiles, and exerted beneficial effects on skeletal muscle. Specifically, the HFD condition was associated with a reduction in the expression levels of 4E-BP1, S6K1, and mTOR genes, whereas the combined interventions were found to elevate the levels of 4E-BP1, S6K1, and IGF-1, in addition to enhancing muscle fiber diameter (53). Another work determined the ramifications of both acute and chronic vitamin C supplementation on brachial artery FMD in both younger and older male subjects, encompassing older individuals who are endurance-trained. Three distinct cohorts were analyzed: young sedentary individuals (*n* = 11, age 25 ± 1 years), older sedentary individuals (*n* = 9, age 64 ± 2 years), and older endurance-trained males (*n* = 9, age 64 ± 2 years). At the initial assessment, normalized FMD was approximately 45% diminished in older sedentary males (0.015 ± 0.001) in comparison to their younger sedentary counterparts (0.028 ± 0.004), while older trained males exhibited preserved levels of FMD (0.028 ± 0.004). An acute intravenous infusion of ascorbic acid led to a substantial elevation in plasma vitamin C concentrations, exceeding 15-fold, and reinstated FMD in older sedentary males (0.023 ± 0.002), with no observable effects in the other cohorts. Chronic oral vitamin C supplementation (500 mg/day for 30 days) exerted no significant influence on FMD within any of the groups, and endothelium-independent dilation responses remained invariant across all groups and interventions (54).

An investigation systematically assessed the impact of swimming exercise in conjunction with silymarin and vitamin C supplementation on liver inflammation, oxidative stress, and histopathological alterations in aged Wistar rats subjected to HFD-induced hepatic injury. A cohort of 40 elderly male Wistar rats was randomly categorized into five distinct groups: control (normal diet), HFD, HFD with supplementation (HFD + Sup), HFD with swimming exercise (HFD + Exe), and HFD with both supplementation and exercise (HFD + Sup + Exe). Following a six-week period of HFD exposure, supplementation was administered via gavage for an additional 8 weeks, while the exercise groups engaged in swimming activities five times per week. The synergistic effect of exercise and supplementation resulted in a marked reduction of liver inflammatory biomarkers, such as tumor necrosis factor-α and interleukin-1β, alongside an elevation in total antioxidant capacity and peroxisome proliferator-activated receptor α (PPAR-α) levels (*p* < 0.05) (55). Recent investigations elucidate the intricate molecular interactions between physical exercise and antioxidant supplementation, notably vitamin C, within various aging models. In geriatric humans and rodents, the integration of physical training with either vitamin C or silymarin effectively modulated oxidative stress, inflammatory signaling pathways, and tissue-specific gene expression profiles. The administration of vitamin C supplementation resulted in a reduction of leukocyte ferritin heavy and light chain (FTH, FTL) mRNA, thereby indicating modifications in iron homeostasis, and exhibited a tendency to diminish IL-6 while concurrently elevating IL-10 mRNA, which reflects its anti-inflammatory properties. In aged rats subjected to liver injury induced by a HFD, the combination of endurance exercise and supplementation augmented antioxidant defenses, elevated the expression of PPAR-α, IGF-1, 4E-BP1, S6K1, and mTOR genes, enhanced skeletal muscle hypertrophy, and mitigated hepatic lipid infiltration. In human subjects, acute intravenous administration of vitamin C reinstated FMD in sedentary older adults, thereby suggesting a link to oxidative stress–mediated endothelial dysfunction. Collectively, these findings substantiate the premise that exercise collaborates synergistically with antioxidant supplementation to modulate redox-sensitive genes, inflammatory pathways, and anabolic signaling mechanisms, thereby enhancing molecular outcomes related to musculoskeletal, vascular, and hepatic health during the aging process.

## 5 Vitamin E and exercise: oxidative stress, muscle damage, and cognitive outcomes

### 5.1 Role as a lipid-soluble antioxidant protecting membranes during eccentric or resistance exercise

Vitamin E plays a crucial role in maintaining the structural and functional stability of cellular membranes, especially when subjected to mechanical stressors such as eccentric or RT. During periods of high-intensity physical activity, the heightened synthesis of ROS may trigger lipid peroxidation within the phospholipid bilayers of muscle and endothelial cells. Vitamin E, predominantly as α-tocopherol, acts by donating electrons to lipid radicals, thereby ceasing the chain reactions of peroxidation and averting membrane damage. This protective mechanism is vital for muscle fibers that experience eccentric contractions, which are inherently susceptible to mechanical disruption and oxidative damage. Furthermore, vitamin E has the capacity to influence intracellular signaling pathways associated with redox homeostasis, inflammation, and apoptosis, thereby enhancing cell viability under exercise-induced stress. Its incorporation into membranes fortifies lipid structures, thereby safeguarding muscle contractile capabilities, mitochondrial integrity, and overall cellular robustness in both physically active individuals and aging populations (56, 57).

### 5.2 Effects on oxidative stress markers, muscle recovery, physical performance, and cognition

Vitamin E supplementation during physical exertion has been correlated with the modulation of oxidative stress and the facilitation of muscular recovery. Empirical research conducted on both human participants and animal models suggests that α-tocopherol diminishes indicators of lipid peroxidation, such as malondialdehyde (MDA), while simultaneously enhancing overall antioxidant capacity subsequent to resistance or eccentric exercise. By mitigating oxidative damage, vitamin E has the potential to alleviate exercise-induced muscular soreness and structural injury, although the evidence regarding direct enhancements in strength and athletic performance remains inconclusive. In addition to its effects on muscle tissue, vitamin E plays a pivotal role in neuroprotection by inhibiting lipid peroxidation within neuronal membranes and bolstering cognitive function under conditions of oxidative stress. Research involving animal models indicates that supplementation can improve spatial memory and diminish neuroinflammation, particularly in models of aging. These results imply that vitamin E operates both locally within muscular tissue and systemically to regulate redox homeostasis, recovery, and cognitive resilience in both physically active individuals and the elderly population (58, 59).

### 5.3 Evidence from clinical and animal studies

Recent randomized controlled trials further support the context-dependent effects of vitamin E supplementation in combination with exercise in aging populations. For instance, a randomized controlled trial investigated the potential benefits of integrating RT with supplementation of vitamins C and E in improving outcomes for older women diagnosed with sarcopenia. A total of 60 participants, aged between 60 and 75 years, were allocated to either an antioxidant supplementation cohort (administering vitamin C at a dosage of 1,000 mg/day and vitamin E at 335 mg/day) or a placebo cohort, with both groups engaging in an identical 12-week elastic-band RT regimen. Although both cohorts exhibited substantial enhancements in muscle mass, strength, and physical performance, the supplementation cohort revealed more pronounced improvements in arm lean mass, skeletal muscle index, handgrip strength, and knee extension strength. No supplementary advantages were noted regarding overall physical performance. On a biochemical level, supplementation significantly augmented redox balance by elevating reduced glutathione levels and diminishing oxidative stress indicators, such as MDA. Inflammatory markers were observed to decrease in both cohorts, with a more significant reduction in IL-6 levels within the supplemented cohort, implying enhanced anti-inflammatory effects (60). Another 12-week randomized investigation aimed to elucidate the effects of RT and vitamin E supplementation (VES) on nonalcoholic fatty liver disease and related metabolic markers in a cohort of 40 adults. Participants were systematically allocated to placebo, VES, RT, or RT + VES groups. The RT regimen comprised eight distinct exercises performed at 60–80% of one-repetition maximum (1RM), executed in three sets of 8–12 repetitions, three times weekly, while VES was administered at a dosage of 800 IU/day. The exercise cohorts exhibited statistically significant enhancements in body composition, lipid profiles, glycemic regulation, and muscular strength in contrast to the non-exercise cohorts. Levels of aspartate aminotransferase and alanine aminotransferase exhibited a decrease across all groups, with the most pronounced reductions observed in the RT + VES cohort. Furthermore, concentrations of CTRP-2 and CTRP-9 diminished in the exercise groups, demonstrating a correlation with the noted metabolic improvements (61). In addition, a research employed a double-blind, randomized controlled methodology to ascertain whether acute supplementation with vitamins C and E mitigates exercise-induced muscle damage (EIMD) among a cohort of 18 endurance-trained runners. Participants consumed either vitamin supplements or a placebo 2 h prior to engaging in 6–8 repetitions of 1 km runs conducted at 75% of their maximum heart rate (HR_max_). Performance metrics, blood lactate levels, and perceived exertion ratings were evaluated immediately following exercise, while creatine kinase (CK) levels, delayed onset muscle soreness, and jump performance were quantified 24 h post-exercise. Immediately after the exercise session, countermovement jump performance exhibited an enhancement across all participants; however, this improvement was sustained at the 24-h mark solely within the vitamin supplementation group. Squat jump performance exhibited an immediate increase in both groups, yet reverted to baseline measurements after 24 h. The elastic index demonstrated an enhancement within the vitamin group; nevertheless, no statistically significant differences emerged between the groups concerning CK levels, lactate concentrations, or other evaluative measures (62).

A study assessed the protective properties of vitamin E supplementation in mitigating exercise-induced oxidative damage among 21 sedentary male participants (comprising nine young individuals and 12 older individuals). The participants were administered either 800 IU of dl-alpha-tocopherol or a placebo daily over a duration of 48 days. The supplementation resulted in a significant elevation of alpha-tocopherol levels in both plasma and skeletal muscle. Following a 45-min bout of eccentric treadmill exercise conducted at 75% of HR_max_, subjects receiving vitamin E supplementation exhibited a markedly lower excretion of urinary thiobarbituric acid-reactive substances compared to those receiving the placebo (12 days’ post-exercise: 35 and 18% above baseline in the young and older supplemented cohorts, respectively, in contrast to 60 and 80% in the placebo group). Participants in the placebo group displayed an increase in muscle lipid peroxidation, a reduction in major fatty acids immediately following exercise, and a trend indicative of elevated conjugated dienes. Notably, plasma levels of vitamins E, C, and uric acid did not demonstrate significant alterations when adjusted for hemoconcentration (63) (Table 3). Another research endeavor delved into the ramifications of a 12-week regimen of vitamin E supplementation (1,000 IU/day) on oxidative stress and muscular impairment induced by eccentric exercise in 16 young (26.4 ± 3.3 years) and 16 older (71.1 ± 4.0 years) healthy male subjects. The participants engaged in downhill running for a duration of 45 min at 75% of their VO₂max both prior to and subsequent to the supplementation period, with blood specimens collected at baseline as well as at 0, 6, 24, and 72 h following the exercise intervention. The exercise regimen resulted in an elevation of serum CK, F₂-isoprostanes (iPF₂α), and MDA across both age cohorts. Additionally, ORAC exhibited a decline at the 72-h mark and demonstrated a correlation with markers of oxidative stress and muscle damage in the younger demographic. The leukocyte 8-OHdG levels remained stable throughout the study. Vitamin E supplementation was observed to mitigate peak CK levels in younger males while concurrently diminishing resting iPF₂α levels and attenuating the increases in 24-h post-exercise iPF₂α in the older male subjects (64).

**Table 3:** Effects of vitamin E supplementation combined with eccentric exercise on oxidative stress, muscle damage, physical performance and cognitive function in older adults.

Study population	Intervention groups	Exercise protocol	Main findings	References
Young (22–29 years) and older (55–74 years) sedentary men	Vitamin E (800 IU/day) Placebo	Downhill running (eccentric), 45 min at 75% HRmax	Plasma & muscle α-tocopherol ↑ (vitamin E) Urinary lipid peroxidation ↓ (vitamin E) Muscle lipid peroxidation ↓ (vitamin E) Muscle fatty acid loss ↓ (vitamin E) Plasma antioxidants ↔ (post-exercise)	(63)
Young (26 years) and older (71 years) healthy men	Vitamin E (1,000 IU/day) Placebo	Downhill running (eccentric), 45 min at 75% VO₂max	CK ↑ (exercise; attenuated by vitamin E in young) Lipid peroxidation markers (MDA, iPF₂α) ↑ (exercise) iPF₂α ↓ (vitamin E at rest and post-exercise in older) ORAC ↓ (72 h post-exercise) DNA damage (8-OHdG) ↔	(64)
Postmenopausal women	Vitamin E (α-tocopherol, 300 mg/day) Exercise (90 min/week) Vitamin E + exercise Control	Walking, 2×/week, 30–60 min/session for 12 weeks	Serum oxidative metabolites ↓ (exercise, vitamin E + exercise) Biological antioxidant potential ↑ (vitamin E, exercise) Plasma thioredoxin ↑ (vitamin E, exercise, vitamin E + exercise)	(65)
Older adults (60–85 years)	Vitamin E (900 IU/day) Exercise (walking 3×/week) Vitamin E + exercise Control	Walking program, 20–50 min/day for 6 months	P3 latency ↑ (exercise groups) Total antioxidant capacity ↔ (vitamin E) Event-related potential amplitude ↔ (all groups) Vitamin E supplementation ↔ cognitive function	(66)
Older sedentary adults (71.5 ± 7.5 years)	Exercise (E) Exercise + vitamin E (EV, 900 IU/day) Vitamin E alone (V) Control (C)	Walking, 3×/week, 70% HRR, 6 months	Body weight ↓ (E, EV) BMI ↓ (E, EV; EV > C/V) 6-min walk ↑ (E, EV; V ↓) Chair stand ↑ (E, EV; V/C ↓) Arm curl ↑ (E, EV) Vitamin E ↔ additive effect beyond exercise	(67)
Male Wistar rats (4–22 months)	Vitamin E supplementation + swim training Swim training alone Sedentary ± vitamin E	Swimming, 30 min/day, 5×/week, 60 days	Swim endurance ↑ (vitamin E + exercise; declines with age) Swim velocity ↔ (no significant change with vitamin E) External work ↑ with age (no vitamin E effect) HDL-C ↑ (vitamin E ± exercise) LDL-C, total cholesterol ↓ (vitamin E) Cholesterol/HDL-C ratio ↓ (vitamin E)	(68)

Symbols indicate the statistical significance of the changes reported in the original studies.

↑/↓: significant increase or decrease (*p* < 0.05).

↔: no significant change (*p* > 0.05).

The purpose of study was to explore the implications of low-volume physical exercise and vitamin E supplementation on oxidative stress among postmenopausal women. The subjects were systematically allocated into four distinct cohorts: control (C, *n* = 8), vitamin E (S, *n* = 8), exercise (Ex, *n* = 6), or a combination of vitamin E and exercise (S + Ex, n = 7). The supplementation regimen included α-tocopherol (300 mg/day) administered over a duration of 12 weeks, whereas the exercise regimen entailed walking for 30–60 min biweekly. Following the 12-week intervention, serum derivatives of reactive oxygen metabolites exhibited a significant reduction in the Ex and S + Ex groups (*p* < 0.05), thereby indicating a decrease in oxidative stress levels. Serum biological antioxidant potential demonstrated an increase in the S and Ex groups, although no such change was observed in the combined group. Furthermore, plasma thioredoxin concentrations were elevated in the S, Ex, and S + Ex groups (*p* < 0.05). These findings imply that even minimal physical activity, with or without the inclusion of vitamin E, has the capacity to enhance resting oxidative stress status and bolster antioxidant defenses in postmenopausal women (65). A randomized controlled trial examined the impact of a six-month regimen of vitamin E supplementation and walking exercise on cognitive functioning among 57 elderly individuals (aged 60–85 years). Participants were allocated into four distinct groups: sedentary control (C), vitamin E (V, 900 IU/day), exercise (E), and a combined intervention of vitamin E plus exercise (EV). The exercise cohorts engaged in progressive walking training three times weekly, achieving a duration of 50 min per session by the eighth week and sustaining this duration for the remainder of the study period. Plasma levels of vitamin E and total antioxidant capacity were assessed, in addition to measuring event-related potentials (P3 latency and amplitude) both prior to and following the intervention. The findings indicated a statistically significant enhancement in P3 latency among the exercise groups, suggesting an improvement in cognitive processing speed. Conversely, vitamin E supplementation, whether administered alone or in conjunction with exercise, did not affect P3 latency or amplitude, thereby suggesting that, within this specific cohort, vitamin E did not exert any influence on cognitive function, despite the cognitive enhancements realized through physical training (66).

A study determined the synergistic effects of a six-month regimen of vitamin E supplementation (900 IU/day) in conjunction with supervised aerobic walking on physical performance metrics and body composition among a cohort of 57 sedentary older adults (mean age 71.5 ± 7.5 years). Participants were systematically allocated into four distinct groups: exercise (E), exercise combined with vitamin E (EV), vitamin E solely (V), or a control group (C). The walking interventions were conducted three times weekly at an intensity corresponding to 70% of the heart rate reserve. The findings indicated that both the E and EV groups exhibited noteworthy reductions in body weight and body mass index (BMI), alongside enhancements in the 6-min walk, chair stand, arm curl, and back scratch assessments. Conversely, the group receiving only vitamin E did not demonstrate any significant improvements in physical performance, with some test outcomes declining. In summary, while aerobic training yielded positive effects on functional capacity and body composition, the addition of vitamin E supplementation did not confer any supplementary advantages beyond those achieved through exercise alone in this demographic (67). Another work examined the implications of vitamin E supplementation on exercise performance and plasma lipid profiles in male Wistar rats categorized into four distinct age groups (4, 8, 12, and 22 months). The rats received oral vitamin E supplementation and were subjected to a swimming regimen of 30 min per day, 5 days per week, over a duration of 60 days. The assessment of exercise performance was conducted via measurements of swim velocity (S(v)), external work (W(ext)), and endurance (E). Notable age-related decrements in swim velocity were identified, particularly among the 22-month-old cohort; however, vitamin E supplementation did not yield statistically significant effects on velocity or external work. Conversely, there was an enhancement in endurance capacity among the vitamin E-supplemented rats, despite a general decline associated with aging. Additionally, plasma cholesterol and LDL-C levels exhibited an upward trend with advancing age, whereas HDL-C concentrations were elevated in all supplemented groups, resulting in a diminished cholesterol/HDL-C ratio (68). Vitamin E plays a critical role in modulating oxidative stress induced by exercise, muscle injury, and metabolic pathways in both aging human subjects and various animal models. The administration of this vitamin elevates α-tocopherol levels in both plasma and skeletal muscle, thereby diminishing lipid peroxidation, MDA, and F₂-isoprostanes, as well as reducing CK release subsequent to eccentric exercise, particularly among younger individuals, while it exerts a partial modulation of oxidative markers in older populations. In postmenopausal females, vitamin E significantly enhances plasma thioredoxin and biological antioxidant capacity, and in older individuals, when combined with either aerobic or RT, it yields improvements in endothelial function, HDL-C concentrations, and endurance capacity without further augmenting performance or cognitive P3 event-related potentials. At the molecular level, vitamin E confers protection to lipid membranes, maintains the integrity of muscle fibers, regulates redox-sensitive signaling pathways, and bolsters antioxidant defenses in the context of mechanical and metabolic stress. Its effects exhibit specificity relative to age and tissue type, implying that vitamin E collaborates with exercise to modulate oxidative, inflammatory, and metabolic gene pathways, thereby sustaining musculoskeletal, vascular, and systemic homeostasis in aging demographics.

## 6 Synergistic effects of vitamins C + E with exercise

### 6.1 Combined antioxidant effects on oxidative stress and inflammation

The amalgamation of vitamins C and E, in conjunction with systematic physical activity, produces synergistic antioxidant and anti-inflammatory outcomes, which are particularly salient in geriatric cohorts. Physical exercise triggers temporary elevations in ROS, deemed essential for adaptive signaling; nevertheless, excessive oxidative stress associated with aging can compromise mitochondrial functionality and exacerbate chronic inflammatory processes. Vitamin C, classified as a water-soluble antioxidant, effectively neutralizes free radicals and reinstates oxidized vitamin E, while vitamin E serves to safeguard lipid membranes against peroxidation (11). When administered alongside moderate exercise, these vitamins contribute to the equilibrium of redox homeostasis without entirely obstructing adaptations induced by physical activity. Empirical investigations reveal that the co-supplementation of vitamins C and E diminishes biomarkers indicative of lipid peroxidation, such as MDA, and mitigates the levels of pro-inflammatory cytokines, including TNF-α and IL-6, in older individuals engaging in exercise training (16, 18). This synergistic interaction may facilitate healthier inflammatory responses, enhance vascular redox signaling, and alleviate age-associated oxidative damage while concurrently preserving the advantageous hormetic effects of physical exercise.

### 6.2 Impact on muscle mass, strength, body composition, and functional outcomes

Exercise remains the foremost intervention to mitigate sarcopenia and functional decline among the elderly population, and its synergistic application with vitamins C and E may further amplify musculoskeletal outcomes. Vitamin C is indispensable for collagen synthesis and the maintenance of muscle ECM integrity, whereas vitamin E serves to safeguard muscle cell membranes from oxidative damage during contraction (17). The integration of resistance and endurance training with antioxidant vitamins has been correlated with enhancements in muscle strength, a reduction in EIMD, and an improved recovery trajectory in older adults (63). Furthermore, vitamin C may augment satellite cell activity and facilitate muscle repair, while vitamin E contributes to the maintenance of neuromuscular function and mitigates fatigue-related oxidative stress. Although excessive supplementation of antioxidants may potentially attenuate anabolic signaling pathways, physiological doses of vitamins C and E seemingly foster lean mass preservation, enhance body composition, and improve functional outcomes (18, 69). This combined strategy may therefore optimize physical performance and independence in aging populations.

### 6.3 Evidence from clinical and animal studies

RT constitutes a fundamental intervention to support healthy aging and prevent sarcopenia, with its effects potentially augmented via prophylactic nutritional strategies. A randomized, placebo-controlled investigation sought to determine whether the integration of RT with antioxidant supplementation confers additional advantages in elderly women diagnosed with sarcopenia. A total of 60 participants, aged between 60 and 75 years, completed a 12-week program of elastic-band RT while receiving either vitamin C (1,000 mg/day) and vitamin E (335 mg/day) or a placebo intervention. Both cohorts exhibited significant enhancements in muscle mass, muscle strength, and physical performance over the duration of the study. Notably, superior improvements were documented in the group receiving antioxidant supplementation, particularly concerning arm lean mass, skeletal muscle mass index, handgrip strength, and knee extension strength. However, advancements in physical performance did not exhibit significant differences between the two groups. Biochemical assessments indicated that antioxidant supplementation led to a pronounced increase in reduced glutathione levels and the GSH/GSSG ratio, alongside a reduction in oxidized glutathione and MDA levels. Markers of inflammation showed a decline in both groups, with interleukin-6 demonstrating a more substantial reduction in the group receiving supplementation (60) (Table 4). Aging is correlated with heightened oxidative stress, which is further intensified in skeletal muscle as a result of physical exercise. An animal study aimed to ascertain whether dietary antioxidant supplementation could enhance muscle functionality and oxidative stress indicators amidst chronic repetitive loading in the context of aging. Young adult and aged Fischer 344 Brown × Norway rats engaged in repetitive stretch–shortening contractions of the dorsiflexor muscles thrice weekly for a duration of 4.5 weeks, with the contralateral limb employed as a control. The animals were administered either a diet enriched with vitamin C and vitamin E or standard chow. Repetitive loading resulted in an enhancement of maximal isometric force and work output in young adult rats, whereas only the positive work parameter exhibited improvement in the supplemented aged rats. Exercise provoked an increase in various oxidative stress biomarkers within the muscle of both age cohorts; however, the administration of vitamin supplementation mitigated these physiological responses. The activities of antioxidant enzymes were differentially influenced by factors such as age, exercise, and supplementation, suggesting a complex regulatory mechanism governing redox adaptations (70).

**Table 4:** Effects of vitamin C and E supplementation combined with resistance/loaded exercise on muscle mass, strength, oxidative stress, and body composition.

Study population	Intervention groups	Exercise protocol	Main findings	References
Older women with sarcopenia (60–75 years)	Antioxidants + RT (vitamin C 1,000 mg/day, vitamin E 335 mg/day) Placebo + RT	Elastic-band resistance training, 12 weeks	Arm lean mass ↑ (AS > PLA) Skeletal muscle mass index ↑ (AS > PLA) Handgrip strength ↑ (AS > PLA) Knee extension strength ↑ (AS > PLA) GSH ↑, GSH/GSSG ratio ↑, GSSG ↓, MDA ↓ (AS > PLA) IL-6 ↓ (AS > PLA), TNF-α ↓ (both groups) Physical performance ↔ between groups	(60)
Aged and young adult rats	Vitamin C + E supplementation Non-supplemented control	Dorsiflexor muscle loading, 3×/week, 4.5 weeks	Positive muscle work ↑ (aged + supplements) Maximal isometric force ↑ (young) Oxidative stress markers (H₂O₂, MDA, 8-OHdG) ↓ (supplemented) Endogenous antioxidant enzymes (MnSOD, CuZnSOD, catalase, GPx) ↑ (age- and exercise-dependent)	(70)
Elderly men & women, mean 65.6 years	Control-placebo RT Vitamin C/E (AS) AS + RT	Resistance training, 6 months	Plasma antioxidants ↑ (AS + RT) Fat-free mass ↑ (AS + RT) Fat mass ↓ (AS + RT) Strength ↔ between groups Pro-oxidative markers ↔	(71)
Elderly adults, mean 74.6 years	Sedentary + Vitamin C/E Aerobic training + Vitamin C/E	Aerobic training, 8 weeks	Plasma vitamin C & E ↑ (~50% & 20%) TBARS ↓, AOPP ↓ (~25% & 20%) Hsp72 expression ↓ (~15%) Training ↔ additional effect beyond supplementation	(72)
Elderly men, 60–81 years	Vitamin C/E (500 mg + 117.5 mg) Placebo	Strength training, 12 weeks	Total lean mass ↑ more in placebo vs. antioxidant (3.9% vs. 1.4%) Muscle thickness (rectus femoris) ↑ more in placebo (16.2% vs. 10.9%) 1RM strength ↑ both groups (15–21%) Vitamin C/E blunted some muscular adaptations	(73)
Elderly men, ~65 years	Sprint interval training (SIT) + placebo or vitamin C 1 g/day + E 235 mg/day	3 weeks, 4–6 × 30 s cycling sprints, 3 sessions/week	Placebo: quinolinic acid ↓, kynurenic acid/quinolinic acid ratio ↑, KAT III ↑ Vitamin C/E: blunted all training effects ↔	(74)
Older adults, 60 years	12-week antioxidant: vitamin E 100 mg, vitamin C 200 mg, beta-carotene 2 mg	Submaximal cycling, 45 min	Plasma α-tocopherol & β-carotene ↑ Exercise-induced hydroxylated antipyrine ratios ↔ TBARS (oxidative stress) ↔	(75)
Elderly men, 67–70 years	12-week resistance training + placebo or vitamin C 1,000 mg + E 235 mg/day	Strength training, 3 sessions/week, 3–15 RM	Placebo: total hip aBMD ↑ 1.0%, lumbar spine aBMD ↑ 0.9%, IGF-1 ↑, leptin ↑, sclerostin ↓ Vitamin C/E: blunted aBMD gains and bone-related benefits ↔/↓	(76)
Healthy elderly men, 60 ± 6 years	Healthy elderly men, 60 ± 6 years	Strength training, 3 months	Serum B12 ↓ and folate ↑ in placebo group Plasma 5-pyridoxal phosphate ↓ in omega-3 and vitamin C/E groups Serum uric acid ↓ only in omega-3 group Reduced aminothiols ↑ in all groups (*p* < 0.001) Redox plasma aminothiol status significantly altered in all groups (*p* < 0.05)	(77)

Symbols indicate the statistical significance of the changes reported in the original studies.

↑/↓: significant increase or decrease (*p* < 0.05).

↔: no significant change (*p* > 0.05).

A research investigation assessed the implications of vitamin C and E supplementation, either in isolation or in conjunction with RT, on oxidative status, muscular strength, and body composition among elderly individuals. A total of 57 participants, comprising both men and women with a mean age of 65.6 years, were allocated to one of four groups: placebo, RT only, antioxidant supplementation (AS), or a combination of AS and RT over a period of 6 months. Initial measurements of fat-free mass and fat mass were found to be consistent across the different groups. Subsequent to the intervention, vitamin E supplementation revealed significant inter-group disparities in body composition, suggesting beneficial impacts on fat-free mass and fat mass. Nevertheless, no substantial improvements in muscular strength were detected among the various groups. Markers of oxidative stress exhibited no alterations in pro-oxidant parameters, whereas an enhancement in plasma antioxidant capacity was particularly noted in the combined AS and RT cohort. These outcomes indicated that although the intake of vitamins C and E, particularly when paired with RT, may bolster systemic antioxidant status, its influence on muscular strength and pro-oxidative markers in the aging population appears to be constrained (71). The aim of study was to elucidate the impact of aerobic training in conjunction with antioxidant supplementation on systemic oxidative stress and leukocyte heat shock protein 72 (Hsp72) expression among older adults. A cohort of 16 individuals aged 70 and above (mean age 74.6 years) were administered daily supplements of vitamin C (500 mg) and vitamin E (100 mg) and were randomly allocated to either a sedentary control group or a tailored aerobic training regimen over a period of 8 weeks. Plasma concentrations of vitamins, aerobic fitness levels, oxidative stress biomarkers, and leukocyte Hsp72 expression were evaluated at baseline and subsequent to graded exercise both prior to and following the intervention. Notable increases in plasma levels of vitamins C and E were observed in both groups, alongside significant decreases in markers indicative of lipid and protein oxidation at both rest and post-exercise intervals. These reductions were found to be consistent irrespective of the participants’ training status. The observed decrease in oxidative stress was complemented by a reduction in Hsp72 expression within monocytes and granulocytes, suggesting a diminished cellular stress response (72).

A randomized, placebo-controlled investigation sought to determine the impact of vitamin C and E supplementation on muscular adaptations resulting from strength training in elderly males. A total of 34 participants, aged between 60 and 81 years, engaged in a 12-week RT regimen while receiving either antioxidant supplementation (comprising vitamin C at 500 mg and vitamin E at 117.5 mg administered before and after training) or a placebo. Body composition was evaluated through dual-energy X-ray absorptiometry (DXA), muscle thickness was measured via ultrasound, and strength was assessed through one-repetition maximum testing. Both experimental groups exhibited significant enhancements in maximal strength, with no discernible differences observed between the groups. Nevertheless, the increases in overall lean mass and rectus femoris muscle thickness were notably more pronounced in the placebo group in comparison to the antioxidant group. The gains in regional lean mass in the trunk and arms, as well as the muscle thickness of the elbow flexors, did not reveal any significant differences between the groups. These results shown that high-dose supplementation of vitamin C and E may inhibit certain hypertrophic adaptations to RT in older males, notwithstanding similar improvements in strength outcomes (73). Another work aimed to ascertain the extent to which antioxidant supplementation modifies exercise-induced adaptations within the kynurenine pathway (KP) among geriatric males. Participants, averaging approximately 65 years of age, engaged in a three-week regimen of sprint interval training (SIT) while receiving either a placebo or vitamin C (1 g/day) and vitamin E (235 mg/day). Blood specimens and muscle biopsies were meticulously collected both prior to and subsequent to the training intervention. In the placebo cohort, SIT was found to diminish circulating concentrations of the neurotoxic metabolite quinolinic acid, concurrently elevating the kynurenic acid-to-quinolinic acid ratio, thereby signifying a transition toward a more neuroprotective KP profile. This phenomenon was associated with augmented skeletal muscle expression of kynurenine aminotransferase III, an essential enzyme implicated in the biosynthesis of neuroprotective metabolites. Conversely, the metabolic and enzymatic adaptations prompted by exercise were not observed in participants who received antioxidant supplementation, indicating that vitamins C and E attenuated the advantageous KP responses to endurance-type exercise in the elderly population (74).

A research sought to ascertain whether a 12-week regimen of antioxidant supplementation alters exercise-induced oxidative stress among older adults. A cohort of 20 participants, approximately 60 years of age, was administered either a supplement comprising vitamin E, vitamin C, and β-carotene or a placebo prior to and following the completion of 45 min of submaximal cycling. The supplementation resulted in a statistically significant elevation of plasma α-tocopherol and β-carotene concentrations, thereby confirming adherence to the supplementation protocol. Nonetheless, markers indicative of exercise-induced oxidative stress, evaluated through antipyrine-derived hydroxyl radical products and thiobarbituric acid reactive substances, exhibited comparable increases in both experimental groups. No significant intra-group or inter-group differences were detected concerning these oxidative stress indices. These results demonstrated that, despite the augmentation of circulating antioxidant levels, supplementation did not mitigate oxidative stress responses to moderate-intensity exercise in the elderly population (75). The objective a study was to determine the impact of high-dose antioxidant supplementation on skeletal adaptations resulting from RT in a cohort of healthy elderly males. A total of 33 participants (approximately aged 67–70 years) engaged in a 12-week supervised strength training regimen while receiving either a placebo or vitamins C (1,000 mg/day) and E (235 mg/day). Within the placebo cohort, areal bone mineral density exhibited an increase at both the total hip and lumbar spine, whereas these enhancements were notably diminished in the antioxidant cohort. Beneficial modifications in bone-related hormones, including elevated levels of insulin-like growth factor-1 and leptin alongside a decrease in sclerostin, were exclusively observed in the placebo cohort. Markers indicative of bone formation exhibited similar increases across both groups, with no observable alterations in bone resorption. These results imply that high-dose antioxidant supplementation may potentially curtail the skeletal benefits induced by exercise in older men (76).

Another investigation examined the impacts of omega-3 fatty acids and the supplementation of vitamins C and E on B-vitamin status and plasma redox aminothiol equilibrium in elderly males participating in strength training. Fifty healthy male participants (average age 60 years) were allocated to receive either a placebo, omega-3 (700 mg/day), or a combination of vitamin C (1 g/day) and vitamin E (235 mg/day) while engaging in a 3-month RT regimen. Strength training induced modifications in B-vitamin profiles in a manner specific to the respective supplements, with notable alterations observed in vitamin B12, folate, pyridoxal phosphate, and uric acid levels across the different groups. Diminished aminothiol concentrations were observed to increase in all groups, signifying a training-associated transition in redox status. Both omega-3 and vitamin C/E supplementation were found to modulate plasma aminothiol redox equilibrium and micronutrient status, indicating that prophylactic nutritional strategies may support redox-related biochemical adaptations to RT and promote healthy aging in older adults (77). The molecular mechanisms that elucidate the interplay between aging, physical exercise, and antioxidant supplementation coalesce around redox-sensitive signaling pathways. Both resistance and endurance training provoke the production of ROS, which serve as critical signals for mitochondrial biogenesis, muscle hypertrophy, collagen remodeling, bone formation, and neuroprotective adaptations. Vitamins C and E play a pivotal role in modulating this process by scavenging excess ROS, maintaining glutathione homeostasis, diminishing lipid peroxidation, and mitigating the effects of inflammatory mediators such as IL-6 and TNF-α. In the context of aged skeletal muscle, antioxidant supplementation has been shown to enhance redox balance and recovery capacity; however, the administration of high doses often attenuates the exercise-induced activation of key regulatory factors such as PGC-1α, NRF2, IGF-1, the suppression of sclerostin, collagen synthesis, and the remodeling of the KP. At the cellular level, the altered post-transcriptional regulation of antioxidant enzymes alongside disrupted ROS-mediated signaling has constrained structural, metabolic, skeletal, and neuroprotective adaptations. Collectively, these findings suggest that while antioxidants are effective in reducing oxidative damage within aging tissues, excessive inhibition of physiological ROS signaling may hinder the molecular adaptations that are essential for exercise-induced resilience in older adults.

## 7 Integrative perspective: collagen, vitamin C, and vitamin E triad

### 7.1 Potential complementary mechanisms and interactions

**Figure 3:**
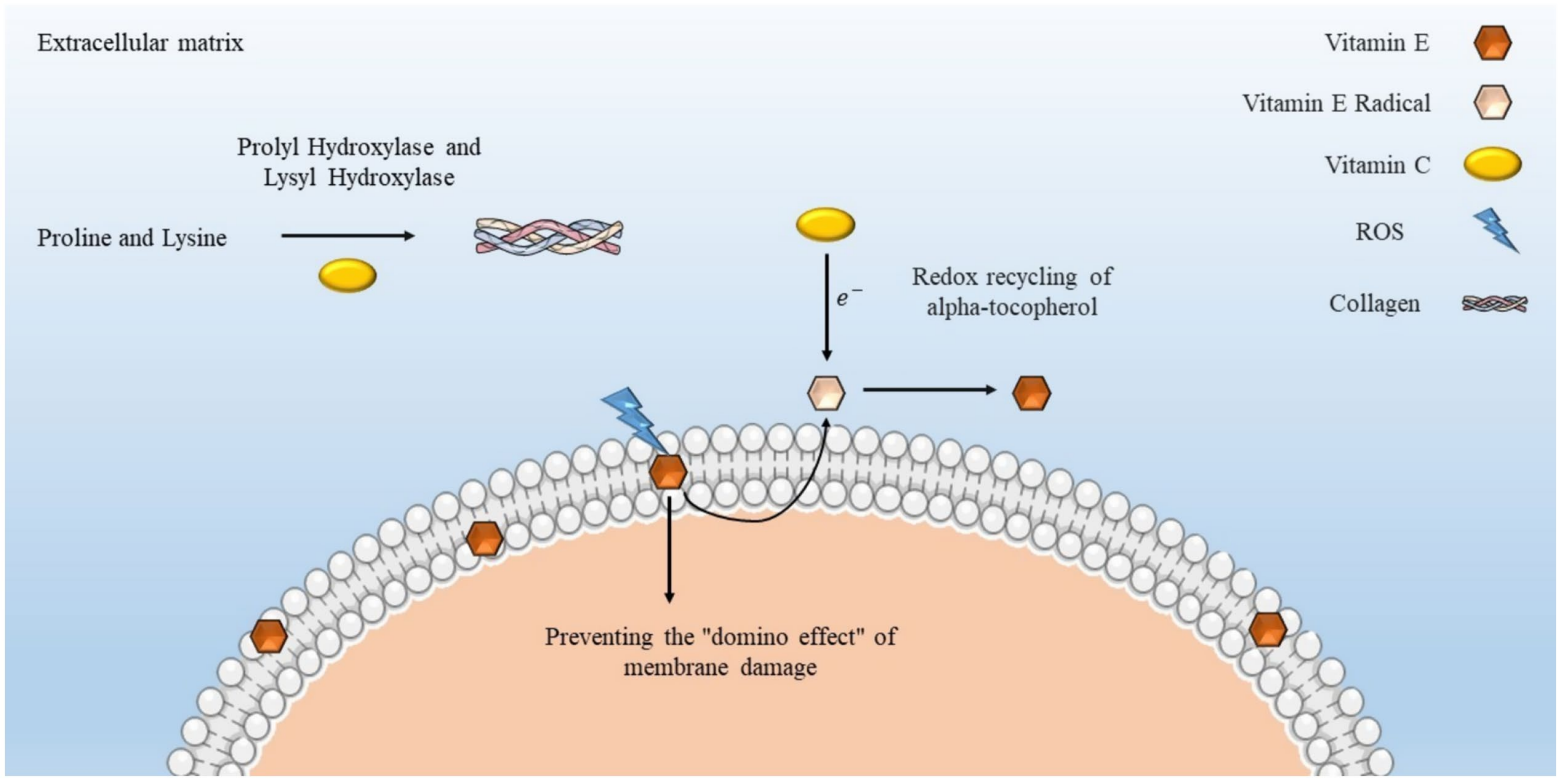
The molecular interaction between the nutritional triad within the cellular and extracellular environments. Vitamin E (tocopherol) is sequestered within the lipid bilayer, where it neutralizes lipid peroxyl radicals. Vitamin C (ascorbate) in the aqueous phase acts as a reducing agent, regenerating the oxidized vitamin E radical back to its active state (redox recycling). Simultaneously, vitamin C serves as an essential cofactor for prolyl and lysyl hydroxylase enzymes, facilitating the hydroxylation of pro-collagen strands. This process ensures the stability and cross-linking of the collagen triple helix, providing a structural scaffold for musculoskeletal and vascular integrity.

Collagen, vitamin C, and vitamin E constitute a functionally interdependent triad that synergistically integrates structural integrity, redox homeostasis, and membrane preservation across senescent tissues. Collagen serves as the ECM framework that is crucial for the transmission of muscle force, vascular elasticity, hepatic architecture, and the scaffolding of neural networks. Vitamin C is essential for the hydroxylation and stabilization of collagen, thus establishing a direct correlation between antioxidant capacity and the remodeling of the ECM as well as tissue repair (8, 78). Concurrently, vitamin E acts to safeguard polyunsaturated fatty acids located within cellular and mitochondrial membranes from the detrimental effects of lipid peroxidation, thereby maintaining cellular integrity in the face of heightened oxidative stress, a condition often encountered during aging and physical exertion (11, 79) (Figure 3). These components engage in interactions at various hierarchical levels. Vitamin C facilitates the regeneration of oxidized vitamin E, thereby perpetuating antioxidant defenses within the lipid phase, while sufficient collagen turnover mitigates the degradation products of the ECM that are pro-inflammatory and have the potential to activate redox-sensitive inflammatory pathways (80, 81). Collectively, this triad reinforces redox equilibrium without negating the physiological signaling of ROS, a critical distinction for the sustenance of adaptive responses. Instead of functioning as discrete supplements, collagen, vitamin C, and vitamin E collaborate as an integrated system that synchronizes structural remodeling with regulated oxidative signaling, which is particularly pertinent in aging tissues characterized by a reduced capacity for repair.

### 7.2 Translational implications for elderly athletes and physically active older adults

For senior athletes and physically active older individuals, the collagen–vitamin C–vitamin E combination presents a practical approach to augment performance resilience rather than solely maximizing performance outcomes. Aging athletes encounter increased vulnerability to connective tissue injuries, protracted recovery periods, endothelial dysfunction, and pronounced inflammatory reactions to training stimuli. Supplementation with collagen, especially when combined with vitamin C, has been demonstrated to enhance the synthesis of collagen in tendons and ligaments, improve joint functionality, and bolster musculoskeletal integrity under mechanical stress (36, 82, 83). Furthermore, vitamin E serves to reduce exercise-induced lipid peroxidation, thereby safeguarding neuromuscular and mitochondrial functionality throughout successive training sessions. Importantly, this triadic framework may facilitate the resolution of the paradox encountered in geriatric populations, wherein physical activity is crucial for physiological adaptation yet concurrently exacerbates oxidative stress and inflammatory responses. By promoting ECM repair, enhancing vascular functionality, and ensuring membrane integrity, the triad could potentially enable older adults to withstand elevated or more consistent training regimens while simultaneously mitigating the risk of injury and expediting recovery processes. This paradigm shift redirects the emphasis from the attenuation of oxidative stress through antioxidant mechanisms to the concept of adaptive buffering, thereby aligning more closely with the pragmatic training requirements faced by aging cohorts.

### 7.3 Considerations for dosage, timing, and type of exercise

The efficacy of the collagen–vitamin C–vitamin E triad is profoundly contingent upon the specific context, thereby necessitating meticulous evaluation of dosage, timing, supplement form, population characteristics, and the modality of exercise. However, it should be noted that robust evidence for the optimal combination of these nutrients with exercise is still limited, as most studies examine single nutrients in isolation, and the reported effects vary across heterogeneous populations. An overabundance of antioxidant intake particularly that of vitamin E, has the potential to attenuate exercise-induced mitochondrial biogenesis and disrupt redox-sensitive signaling pathways, including PGC-1α and NRF2 (17, 18). Consequently, it is advisable to utilize physiological rather than pharmacological dosages, particularly when integrated with endurance training. Timing holds equal significance in this context. The efficacy of collagen and vitamin C appears to be maximized when ingested prior to exercise or in immediate temporal relation to mechanical loading (84), thereby enhancing collagen synthesis in response to exercise-induced signaling pathways. The form of supplementation (e.g., hydrolyzed vs. gelatinized collagen, ascorbic acid vs. buffered vitamin C) further modulates bioavailability and physiological impact. Nevertheless, the magnitude of these effects, especially in older adults, remains to be fully confirmed by longitudinal and adequately powered trials that account for individual variability in age, nutritional status, and baseline physical activity. Conversely, vitamin E may be optimally administered away from training sessions or utilized intermittently during periods characterized by elevated oxidative stress, such as intensified training regimens or recovery phases. Resistance and impact-oriented exercises are likely to receive greater advantages from collagen-centric supplementation, while endurance and mixed modality training necessitate a more judicious antioxidant approach. Given the current evidence, all recommendations should be interpreted as prophylactic and preventive strategies rather than established therapeutic protocols for sarcopenia or other age-related disorders. Consequently, individualized prophylactic strategies, tailored to training status, chronological age, baseline nutritional sufficiency, supplement form, exercise modality, and the current gaps in mechanistic evidence, are essential to optimize adaptive responses and promote healthy aging.

## 8 Clinical and practical implication

The amalgamation of collagen, vitamin C, and vitamin E supplementation with systematically structured exercise protocols presents a compelling approach to the promotion of healthy aging through preventive nutritional strategies and the preservation of musculoskeletal, metabolic, vascular, immune, and cognitive health within geriatric populations. The daily consumption of 10–30 g of hydrolyzed collagen peptides, particularly those enriched with glycine, proline, and hydroxyproline, has the potential to augment MPS, enhance tendon resilience, and maintain joint integrity, particularly when ingested immediately prior to or following resistance or endurance training. Vitamin C supplementation in the range of 500–1,000 mg/day bolsters antioxidant defenses, modulates inflammatory responses, and amplifies immune-related gene expression, thereby complementing exercise-induced adaptations in both vascular and hepatic functions. Vitamin E, administered at doses of 200–400 mg/day, mitigates lipid peroxidation while diminishing exercise-induced oxidative stress, particularly during high-intensity or eccentric training; nevertheless, prolonged administration of elevated doses may attenuate adaptive responses, which encompass exercise-induced improvements in muscle hypertrophy and bone integrity. RT conducted two to three times weekly, with a focus on the major muscle groups, when complemented by this triad of supplementation, consistently supports improvements in muscle hypertrophy, strength, and functional performance as part of a preventive strategy rather than a therapeutic intervention for established sarcopenia, and with caution in extrapolating short-term biomarker changes to long-term functional outcomes. Conversely, aerobic or endurance training is instrumental in enhancing vascular, metabolic, and hepatic health outcomes. It should be emphasized that the physiological impact of supplementation is influenced by the choice of supplement form, timing relative to exercise, and individual population characteristics, including age, baseline nutritional status, comorbidities, and habitual physical activity level. It is posited that low-to-moderate intensity training, when paired with antioxidant supplementation, may be particularly efficacious for frail or sedentary older adults, while high-intensity exercise necessitates vigilant monitoring to avert the risk of injury. Therefore, all recommendations should be interpreted as individualized, preventive strategies aimed at maximizing adaptive responses while ensuring safety, rather than as universally applicable therapeutic protocols. The choice of supplement form, timing relative to exercise, and individual population characteristics (age, baseline nutritional status, comorbidities, and physical activity level) further modulate the physiological impact and efficacy of interventions. The formulation of supplementation protocols should take into account the individual’s baseline nutritional status, pre-existing comorbidities, and the presence of polypharmacy, as well as the potential for interactions, particularly with anticoagulants or fat-soluble medications, which require thorough evaluation. Customized, prophylactic strategies, including the assessment of oxidative stress markers, muscle functionality, and metabolic profiles, are recommended to maximize benefits for healthy aging and ensure participant safety. The integration of dietary interventions with structured exercise regimens presents a pragmatic, evidence-based methodology to maintain or enhance systemic health and functional independence in older adults through preventive nutritional and lifestyle approaches, with particular emphasis on those who are physically active or engaged in wellness programs.

## 9 Limitations and future directions

Despite robust empirical evidence substantiating the synergistic interactions between collagen, vitamin C, and vitamin E supplementation in conjunction with physical exercise among geriatric populations, considerable limitations persist that necessitate meticulous scrutiny. Numerous extant investigations are hampered by restricted sample sizes, short-term intervention durations, and limited follow-up, which complicates the extrapolation of short-term biomarker changes to long-term functional outcomes. Additionally, there is a lack of uniformity in participant characteristics, encompassing variations in age, baseline nutritional status, comorbid conditions, sex, and habitual physical activity levels, which further challenges the generalizability of findings. The inconsistency in supplementation protocols, which includes variations in dosage, formulation, timing relative to exercise, and bioavailability, further complicates the comparative analysis of results across different studies. Likewise, the heterogeneity of exercise interventions, which spans modalities such as RT, BFR, endurance training, and eccentric exercise protocols, contributes additional variability in outcomes, thereby complicating the development of generalizable recommendations for preventive healthy aging rather than treatment of established sarcopenia. The mechanistic comprehension of the subject matter remains incomplete; the influence of antioxidant supplementation on exercise-induced signaling cascades, encompassing MPS, mitochondrial adaptation, redox-sensitive pathways, inflammatory modulation, and immune responses, continues to be insufficiently investigated. Moreover, most studies examine single nutrients in isolation rather than the integrated collagen–vitamin C–vitamin E triad, which limits mechanistic clarity across musculoskeletal, metabolic, vascular, hepatic, immune, and cognitive systems. Some empirical evidence suggests that high-dose antioxidants may diminish specific adaptive responses to exercise; however, these interactions are not thoroughly elucidated within geriatric populations. Future research should prioritize well-powered, longitudinal, randomized controlled trials with multi-omic approaches (metabolomics, proteomics, genomics) and sophisticated biomarkers of oxidative stress, inflammation, immune functionality, and muscle-tendon adaptation. Studies should also stratify participants by age, baseline nutrition, training status, sex, and comorbidity profile to understand differential responses and optimize preventive outcomes. Furthermore, nascent research should examine the synergistic effects of combined nutraceutical interventions, optimal dosing regimens, timing relative to exercise, and precision-targeted exercise prescriptions to enhance musculoskeletal, metabolic, vascular, hepatic, immune, and cognitive outcomes in aging populations, with a clear focus on prophylactic, preventive strategies rather than therapeutic interventions for established sarcopenia or age-related disease.

## 10 Conclusion

The accumulated body of evidence emphasizes the synergistic efficacy of collagen, vitamin C, and vitamin E supplementation when integrated with structured exercise regimens as a preventive strategy for healthy aging rather than as a therapeutic intervention for established sarcopenia. Collagen plays a pivotal role in the preservation of skeletal muscle mass, enhancement of strength, and maintenance of connective tissue integrity, whereas vitamin C plays a crucial role in bolstering antioxidant defenses, facilitating collagen synthesis, and modulating immune responses. Vitamin E further augments resilience to oxidative stress, mitigates muscle damage induced by exercise, and potentially contributes to the enhancement of vascular and cognitive functionalities. When this triad is incorporated with RT, endurance training, or BFR protocols, it manifests additive benefits across multiple systems, including musculoskeletal, metabolic, vascular, hepatic, immune, and cognitive domains, highlighting the integrative, multi-system nature of these interactions. Nevertheless, the outcomes of interventions are contingent upon various factors including dosage, timing, formulation, baseline nutritional and health status, as well as the modality of exercise, thereby underscoring the imperative for individualized, context-specific strategies. Collectively, the existing empirical evidence substantiates the methodical application of this triad as a prophylactic, non-pharmacological approach aimed at enhancing adaptive responses to exercise, mitigating age-associated functional decline, and promoting overall healthspan. Healthcare professionals, exercise physiologists, and nutritionists should contemplate the integration of customized supplementation with carefully designed, exercise-integrated preventive protocols to optimize musculoskeletal performance, maintain metabolic homeostasis, ensure vascular integrity, bolster immune competence, and enhance cognitive health in the aging population.
